# Improving drug delivery strategies for lymphatic filariasis elimination in urban areas in Ghana

**DOI:** 10.1371/journal.pntd.0005619

**Published:** 2017-05-11

**Authors:** Nana-Kwadwo Biritwum, Bertha Garshong, Bright Alomatu, Dziedzom K. de Souza, Margaret Gyapong, Dominique Kyelem

**Affiliations:** 1 Neglected Tropical Diseases Program, Ghana Health Service, Accra, Ghana; 2 Research and Development Division, Ghana Health Service, Accra, Ghana; 3 Noguchi Memorial Institute for Medical Research, University of Ghana, Legon-Accra, Ghana; 4 Dodowa Health Research Center, Dodowa, Ghana; 5 Taskforce for Global Health, Decatur-Atlanta, Georgia, United States of America; Task Force for Child Survival and Developmentorce for Global Health, UNITED STATES

## Abstract

The Global Program to Eliminate Lymphatic Filariasis (GPELF) advocates for the treatment of entire endemic communities, in order to achieve its elimination targets. LF is predominantly a rural disease, and achieving the required treatment coverage in these areas is much easier compared to urban areas that are more complex. In Ghana, parts of the Greater Accra Region with Accra as the capital city are also endemic for LF. Mass Drug Administration (MDA) in Accra started in 2006. However, after four years of treatment, the coverage has always been far below the 65% epidemiologic coverage for interrupting transmission. As such, there was a need to identify the reasons for poor treatment coverage and design specific strategies to improve the delivery of MDA. This study therefore set out to identify the opportunities and barriers for implementing MDA in urban settings, and to develop appropriate strategies for MDA in these settings. An experimental, exploratory study was undertaken in three districts in the Greater Accra region. The study identified various types of non-rural settings, the social structures, stakeholders and resources that could be employed for MDA. Qualitative assessment such as in-depth interviews (IDIs) and focus group discussions (FGDs) with community leaders, community members, health providers, NGOs and other stakeholders in the community was undertaken. The study was carried out in three phases: pre-intervention, intervention and post-intervention phases, to assess the profile of the urban areas and identify reasons for poor treatment coverage using both qualitative and quantitative research methods. The outcomes from the study revealed that, knowledge, attitudes and practices of community members to MDA improved slightly from the pre-intervention phase to the post-intervention phase, in the districts where the interventions were readily implemented by health workers. Many factors such as adequate leadership, funding, planning and community involvement, were identified as being important in improving implementation and coverage of MDA in the study districts. Implementing MDA in urban areas therefore needs to be given significant consideration and planning, if the required coverage rates are to be achieved. This paper, presents the recommendations and strategies for undertaking MDA in urban areas.

## Author summary

The control of lymphatic filariasis depends on the treatment of entire endemic communities, ensuring that a greater proportion of the population is treated. In urban areas, this can be very difficult to achieve. In Ghana, parts of the Greater Accra Region, where the capital city is located, are also endemic for lymphatic filariasis. Treatment in these areas started in 2006, but the proportion of people treated has continuously been below the required programmatic coverage levels. To understand the reasons behind this, a study was undertaken in three endemic districts. The study was carried out in three stages; pre-treatment, treatment and post-treatment. Individuals and groups of people were interviewed in the pre-treatment phase, following which their concerns were used to plan and execute treatment activities. After treatment, some individuals were again interviewed to assess the effectiveness of the interventions. The results showed that the knowledge and behavior of community members towards the disease and treatment activities improved from the pre-treatment to the post-treatment reviews. Many factors were identified including, financial, management and leadership issues that should be considered when planning treatment activities in urban areas.

## Introduction

Lymphatic Filariasis (LF) is a significant health problem in many developing countries with over 1 billion people believed to be at risk in endemic areas [1,2]. LF is also the second leading cause of permanent disability after leprosy [3] and undermines the social and economic welfare of affected people and communities [4]. The World Health Assembly passed a resolution in 1997 to eliminate LF by the year 2020. In the year 2000, the World Health Organization launched the global programme to eliminate LF [5]. The strategy employed involves annual mass treatment with single-dose diethylcarbamazine (DEC) or Ivermectin (IVM) in combination with Albendazole (ALB) for 4–6 years. This is the principal strategy of LF elimination. The strategy is backed by studies that have shown that one or two annual treatments with antifilarial drugs exert only limited effects on microfilaria rates and intensities and multiple rounds of treatment are necessary to reduce the microfilaria prevalence to zero [6].

Drug distribution in urban areas, however, has become a major challenge for programs involved in the elimination of LF [2,7]. An adequate level of 65% epidemiologic coverage is needed to eliminate LF [8], but this continues to remain a challenge in urban areas for most countries including Ghana. While there is little literature available on urban MDA [9–11], the scarcity of information makes it even more difficult to solve the challenges presented. The Greater Accra Region (GAR) with Accra as the capital city of Ghana continues to get low coverage for its annual MDA. Accra Metropolitan district started treatment in 2006 and had a fluctuating epidemiologic coverage of 49.4%, 11.1%, 37.6% and then 60.2% respectively for 2006, 2007, 2008 and 2009 treatments.

Urban areas are generally known to have a mix of diverse populations. They also tend to have very densely populated urban slums with large mobile populations. This phenomenon requires varied but specific strategies tailored for the different identifiable groups rather than a uniform distribution strategy. The design of interventions for specific groups in urban areas requires appropriate diagnosis of the problem. Thus, in the Greater Accra region there was need to identify the reasons for poor treatment coverage in order to design specific strategies to improve the delivery of MDA. As such, the main objective of this study was to identify the opportunities and barriers for implementing MDA in urban settings in order to develop appropriate strategies for MDA in these urban settings.

## Method

### Ethical approval

Approval for the study was received from the Ethics review Committee of the Ghana Health Service. Written consent was obtained from all individuals who participated in the study.

#### Study area

The study was carried out in the Greater Accra region. It is the smallest of the 10 administrative regions, occupying a total land surface of 3,245 square kilometers or 1.4 per cent of the total land area of Ghana. In terms of population, however, it is the second most densely populated region, with a projected population of 4,368,351 in 2011 and population density of 1,346 persons per square kilometer. The region has an annual population growth rate of 3.1% per annum, which is higher than the national average of 2.5% per annum for the country (2010 Census).

There are 10 administrative metropolitans, municipal or district assemblies in the region. LF is endemic in 5 of the 10 areas and these are the Accra Metro, Ga East, Ga West, Ga South and Ledzokuku Krowor Municipalities (www.ghanadistricts.com). This study was conducted in the Ledzokuku Krowor Municipal area, Ayawaso sub metropolitan district and Ashiedu Keteke sub-metropolitan district.

The Ayawaso sub-metropolitan area is small but densely populated, with a projected population of 562,921 for 2011. It has a variety of ethnic groups, and other nationals from neighbouring countries like Burkina Faso, Niger, Mali, Togo and Nigeria among others. Settlements in the sub-metro area can be classified into high income and low income areas, including one of the major slums in the city.

The Ashiedu Keteke sub-metro area is the smallest yet the most densely populated in the Accra metropolis. It has a projected population of 148,735 for 2011. It is at the central business district. The Ashiedu Keteke sub-metro zone has the largest slum in the city of Accra (Sodom and Gomorrah), which started as a temporary settlement area for people who were fleeing the war in the Northern part of Ghana (endemic for LF) in the 1990’s.

The Ledzokuku Krowor Municipal area was created out of the Accra metro in 2008. It has a projected population of 383,465 for 2011. The housing environment is characterized by haphazard development, inadequate housing infrastructure, poor drainage, poor roads, erosion and high population concentrations.

#### Methodology

This is an experimental, exploratory study. Sampling relied on the identification of socio-economic groups, the social structures, stakeholders and resources that exist in the study areas that could be employed for MDA. A mixed method sampling of purposive and random selection was used, depending on the tool employed. The study was done using mainly qualitative methods such as in-depth interviews (IDIs) and focus group discussions (FGDs) with community leaders, community members, health providers, NGOs and other stakeholders in the community. IDIs and FDGs were only implemented during the pre-intervention phase. Observation, transect walks and community maps were undertaken and documented with community members to provide a demarcation of the communities within which to implement specific strategies with the involvement of the population. In addition a quantitative approach based on a random selection of houses for household surveys was employed to assess MDA coverage.

The study was undertaken in three phases: pre-intervention (5 months), intervention (3 months) and post-intervention (8 months) which assessed the profile of the urban areas and identified reasons for poor treatment coverage using both qualitative and quantitative tools. The pre-intervention phase was carried out in all three districts, and assessed the MDA undertaken in December 2010. It was undertaken to inform the design and implementation of appropriate interventions. The intervention phase involved the designing and testing of urban MDA using information from the pre-intervention phase. Information about the different groups within the urban settings and suggestions from the various stakeholders and community members on the appropriate ways of implementing MDA in urban settings were used in the design of appropriate strategies. The intervention was implemented in two of the districts/sub-metropolitan districts (Ledzokoku Krowor and Ashiedu Keteke), while Ayawaso was used as a control district where no new interventions were implemented. The post-intervention phase involved an evaluation of how the implementation of the new interventions in urban settings had worked, and assessed the MDA undertaken in March 2012.

#### Training and pretesting

Research assistants were selected and trained to carry out the structured interviews, FGDs, IDIs, observations and assist the population to map out their community. Data collection tools were pre-tested in a different LF endemic urban area. Local language translations of all study tools except those for the health workers were provided. The local languages were Ga and Twi, the predominantly spoken languages in the Greater Accra region. Back translation into English as a check on the accuracy of the translation was done.

#### Focus group discussions

Identification of members of the FGD was done with the assistance of community members and any social groupings available within the selected urban areas. All focus groups were homogeneous with regard to sex. Each FGD was made up of 6 to 8 participants, willing to take part in the study, and lasted for an hour. A total of four FGDs (a minimum of one FDG in each of the social strata or groupings) were conducted with community members in each of the three study districts to solicit information on a description of existing programs and health delivery outlets (both public and private) that were acceptable to the different groups in these settings, and suggestions from the community on appropriate health service delivery points and their participation were sought. Discussions focused on LF, its cause, presentation, control, prevention and the risk perception with regard to individuals and the community in general. Awareness about the LF elimination programme with emphasis on MDA was assessed (See Supplementary S1 File).

#### In-depth interviews

A total of about 40 in-depth interviews were conducted with representatives of health workers, religious leaders, traditional authorities, Non-Governmental Organizations (NGOs) and drug distributors. Stakeholders in the IDIs provided information on the types of program and health service delivery outlets existing in their settings. Their experiences and challenges in supporting programs in the area were assessed. The IDIs provided information on the capacity and processes involved in the implementation of MDA on training, social mobilization, demarcation of communities for the distribution of drugs and methods for undertaking monitoring and supervision (Supplementary S2 File).

#### Household surveys

Trained facilitators visited identified communities to make initial contact with potential study participants and explained the purpose of the study in order to get them to be part of the discussion. Thirty clusters of the smallest units in the selected urban areas in each of the 3 selected districts/sub-metro areas had their adult populations interviewed using the structured questionnaires. Three urban areas were randomly selected from the 3 endemic districts. Each of the urban areas was further divided into townships. Each of these townships was then stratified into low income, middle income and high income areas. The low income areas were further sub-divided into the indigenous and non-indigenous populations. A list of the smallest units of these townships was compiled. For each of the lists, 7 households were randomly selected. The selection of the 7 households was based on the EPI 7/30 cluster sampling method [12]. About 60% of the clusters were randomly selected from the low-income areas while the remaining 40% were randomly selected from the middle-income and high-income areas. The low income populations tend to be more densely populated than the middle and high income populations and therefore it was decided to interview more households in each of the low income populations as compared to the middle and high income populations. For each of the selected households, an adult over 18 years was interviewed, at the pre-intervention stage. A structured questionnaire was designed and applied for these interviews (Supplementary S3 File). During the post-intervention evaluation, all adults above the age of 18 years present in the house at the time of the survey were interviewed.

#### Data management

*Qualitative data* were organized and managed using MAXQDA software. Qualitative data were transcribed from the local language to English. The transcriptions were coded using themes that corresponded to the issues under study. A sample of the tapes was listened to and checked for the accuracy of transcriptions. *Quantitative data* were doubly entered, cleaned and consistency checks performed. Data were entered using CSpro and Epi Info. Data cleaning, validation and analysis were done using a combination of MS Excel, Access, and Epi Info. The relationship between variables was presented graphically and critically analysed to ensure clarity and accuracy. The data were presented, with percentages calculated to assess the impact of the interventions. For the purposes of evaluating the impacts of the interventions, the pre and post-intervention results were tabulated together.

## Results

### Pre and post intervention evaluations

#### Demographic characteristics of respondents

**Fig 1:**
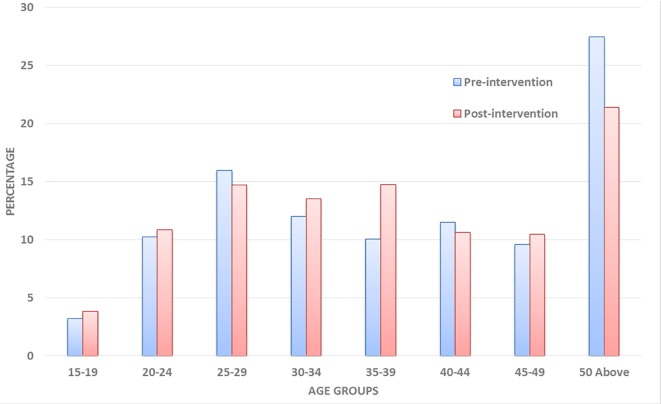
Age and sex distribution of the study populations.

For the household survey, a total of 644 respondents were interviewed during the pre-intervention surveys and 630 during the post-intervention surveys, distributed in 15 zones and three (3) sub-metro areas. Majority of the respondents in both surveys were within the 25–40 age groups (Fig 1). Table 1 presents the demographic information about the study respondents. More females (64.6% and 71.6% respectively for pre and post-intervention surveys) were interviewed compared to males (35.4% and 28.4% for pre- and post-intervention surveys). Majority of respondents had middle/junior secondary education. The number of people with tertiary education was much higher in the post-intervention survey (9.2%) compared to the pre-intervention survey (0.5%). In terms of occupation, there was not much difference between the pre and post-intervention assessments, except for the number of artisans interviewed during the post-intervention survey.

**Table 1:** Demographic characteristics of respondents.

Indicator	Pre-intervention (N = 644)	Post-intervention (N = 630)
**Sex**		
Female	416 (64.6%)	451 (71.6%)
Male	228 (35.4%)	179 (28.4%)
**Marital status**		
Single	202 (31.4%)	364 (57.8%)
Married	383 (59.5%)	42 (6.7%)
Divorced	37 (5.7%)	183 (29.0%)
Widowed	22 (3.4%)	41 (6.5%)
**Level of Education**		
None	65 (10.1%)	65 (10.3%)
Primary	84 (13.0%)	89 (14.1%)
Middle/JSS	243 (37.7%)	281 (44.6%)
SHS/ Secondary	144 (22.4%)	96 (15.20%)
Commercial/Vocational/Technical	101 (15.7%)	39 (6.2%)
Tertiary	3 (0.5%)	58 (9.2%)
Non Formal	4 (0.6%)	2 (0.3%)
**Occupation**		
Trader	284 (44.1%)	287 (45.6%)
None	96 (14.9%)	99 (15.7%)
Artisan	78 (12.1%)	178 (28.3%)
Farmer	2 (0.3%)	2 (0.3%)
Fisherman	4 (0.6%)	6 (1.0%)
Public Servant	28 (4.3%)	30 (6.0%)
Teacher	10 (1.6%)	13 (2.1%)
Other	142 (22.0%)	7 (1.1%)
**Religion**		
Christian	566 (87.9%)	551 (87.5%)
Moslem	64 (9.9%)	63 (10.0%)
No Religion	10 (1.6%)	13 (2.1%)
Traditional	3 (0.5%)	3 (0.5%)
Other	1 (0.2%)	0 (0.0%)

#### Knowledge and causes of lymphatic filariasis

Respondents were asked questions about both elephantiasis and hydrocele. During the pre-intervention survey, 96.3% of respondents had heard about elephantiasis. On the other hand 61.3% had heard about hydrocele (Table 2). In the post-intervention surveys, 97.4% had heard of elephantiasis, whiles only 49.5% heard of hydrocele. The lack of difference in the pre and post-intervention knowledge of hydrocele responses can be attributed to the responses obtained from Ashiedu Keteke and Ledzokuku during the post-intervention surveys.

**Table 2:** Knowledge of lymphatic filariasis.

Sub Metro	Ashiedu Keteke		Ledzokuku		Ayawaso (Control)		Overall	
Intervention	Pre (N = 213)	Post (N = 210)	Pre (N = 214)	Post (N = 210)	Pre (N = 217)	Post (N = 210)	Pre (N = 644)	Post (N = 630)
Elephantiasis	206 (96.7%)	206 (98.0%)	209 (97.7%)	203 (96.6%)	205 (94.5%)	205 (97.6%)	620 (96.3%)	614 (97.4%)
Hydrocele	136 (63.4%)	70 (33.3%)	124 (57.9%)	108 (51.4%)	135 (62.2%)	134 (63.8%)	395 (61.3%)	312 (49.5%)

With respect to knowledge of the cause of filariasis, it was clear from the responses that there were misconceptions about what causes LF (Table 3). Several causes of the disease were mentioned including; mosquito bites, playing in the mud, drinking bad water, living close to rivers, living in dirty surroundings, walking bare foot in the rain, stagnant water with insects, cold weather conditions, poor personal hygiene, and eating foods that one is allergic to or eating unhealthy and sugary food. The number of respondents who mentioned mosquito bites as a cause of the disease improved from the pre-intervention (13.0%) to the post-intervention (21.0%) assessments. Similarly, the responses with reference to germs also increased from the pre to the post-intervention. Others believed the germ that causes the disease was created in the laboratory and later a cure found for it by the same people who created the disease. Some quotes following FGDs are below.

-*The disease they say is caused by the elephant’s faeces*. *They say when it defecates and someone steps on the faeces the person will get the disease*. *That is what I heard from my grandfather (FGD*, *community member)*.

-*I have seen that*… *to you someone can put medicine (charm) on the ground and you step on it that can sometimes bring the disease (FGD males)*.

-What I know is that it is a germ that is in the water and when you step into the water, the germ enters your leg, then it will stay in your leg and it will be eating into the leg until the leg starts to swell (FGD, community member).

-*They made us understand that a type of mosquito brings the disease*. *If I have the disease and the mosquito bites me and sucks my blood and bites you*, *after sometime*, *let’s say six months or in a years’ time it will start to develop in you too (FGD*, *community male)*.

**Table 3:** Means of contracting elephantiasis.

Means	Pre-Intervention (N = 644)	Post-Intervention (N = 630)
**Don't Know**	328 (50.9%)	296 (47.0%)
**Drinking contaminated water**	137 (21.3%)	115 (18.3%)
**Mosquito Bite**	84 (13.0%)	132 (21.0%)
**Germs**	31 (4.8%)	121 (19.2%)
**Eating Contaminated food**	27 (4.2%)	54 (8.6%)
**Hereditary**	18 (2.8%)	18 (2.9%)
**Juju/Witchcraft**	17 (2.6%)	11 (1.8%)
**Other**	78 (12.1%)	-

There is a low risk perception of contracting elephantiasis–the symptom of the disease. Majority of those interviewed indicated that they were not at risk (Table 4), indicating a lack of understanding of the development of the disease. Overall, the risk perception decreased from the pre-intervention (27.9%) to the post-intervention (24.8%). In Ashiedu Keteke, the risk perception during the post-intervention assessment (41.3%) was higher compared to the pre-intervention values (26.2%), and can be attributed to the health education provided during the intervention. Related to risk perception is whether treatment is available or not for elephantiasis. During the pre-intervention most respondents did not know of the availability of any treatment for elephantiasis. Only 130/613 (21.2%) individuals indicated treatment was available for elephantiasis. 483/613 of the respondents (78.8%) said that either they did not know if treatment for elephantiasis was available or indicated that treatment was not available. In terms of knowledge of availability of treatment for hydrocele 111/395 (28.1%) of respondents knew about the availability of treatment for hydrocele, 116/395 (29.4%) did not think that there was any treatment available for individuals with hydrocele.

**Table 4:** Risks for contracting elephantiasis.

Sub Metro	Yes (Pre-Intervention)	Yes (Post-Intervention)
**Ashiedu Keteke**	54 (26.2%)	86 (41.3%)
**Ledzokuku**	53 (25.4%)	35 (17.1%)
**Ayawaso (Control)**	66 (32.2%)	32 (15.6%)
**Total**	173 (27.9%)	153 (24.8%)

Respondents were asked how LF can be prevented. A little over one third (36%) of the respondents did not know whether LF was preventable or not. There was an overall improvement in the responses provided by the study respondents (Table 5). The number of people who indicated taking drugs as a prevention measure increased from 25.9% to 40.5% from the pre to the post-intervention assessments. Similarly, sleeping in mosquito nets (7.0% to 37.1%) and keeping the environment clean (20.7% to 49.2%) as control measures also improved from the pre to the post-intervention assessments. The responses categorized as ‘other’ include not drinking contaminated water, not walking in contaminated water, avoiding insects/snake bites, environment/water body spraying, frequent hospital check-ups, eating good balanced diet, health education and avoiding juju and herbs.

**Table 5:** Knowledge about LF prevention.

Sub Metro	Ashiedu Keteke		Ledzokuku		Ayawaso (Control)		Overall	
Intervention	Pre (N = 213)	Post (N = 210)	Pre (N = 214)	Post (N = 210)	Pre (N = 217)	Post (N = 210)	Pre (N = 644)	Post (N = 630)
Taking Drugs	25.4%	51.4%	27%	18.1%	25.1%	51.4%	25.9%	40.5%
Sleeping Mosquito Net	4.2%	12.4%	6.6%	11.0%	10.0%	12.4%	7.0%	37.1%
Keeping Environment Clean	20.3%	41.4%	19.3%	22.4%	22.3%	41.4%	20.7%	49.2%
Don't Know	40.7%	0.3%	37.7%	52.9%	29.9%	0.3%	36.0%	26.2%
Other	9.3%	0.0%	9.4%	0.0%	12.7%	0.0%	10.5%	0.0%

Several prevention strategies were mentioned during the FGDs. These include boiling or treating water before drinking, desisting from using water that domestic animals drink from, keeping the environment clean, spraying gutters, proper disposal of waste, good ventilation of rooms, living a healthy life and eating healthily, good personal hygiene, buying only prescribed medication, washing hands before eating, prayers, sleeping under a treated bednet, regular medical check-ups, and taking the drugs that are distributed. Below are some narratives (quotes) on the prevention of the disease.

-*It is all about cleanliness*, *we should keep our surroundings clean and we must keep ourselves clean (FGD*, *Community member)*.

-*The government can help by spraying the gutters*. *You have to pay to dispose of rubbish and people litter around*. *(FGD*, *Woman)*.

Regarding the prevention of LF, majority of the responders (54.0% pre-intervention) indicated lack of awareness of the services Ghana Health Service provides for people with LF. This however decreased to 41.9% post-intervention (Table 6). The knowledge of health services provided by the Ghana Health Service increased from the pre-to the post-intervention assessment. While only 0.2% knew of the distribution of mosquito nets in the pre-intervention assessment, this increased considerably to 19.8% in the post-intervention survey.

**Table 6:** Knowledge on available health service support for LF patients by sub-metro.

Sub Metro	Ashiedu Keteke		Ledzokuku		Ayawaso (Control)		Overall	
Intervention	Pre (N = 213)	Post (N = 210)	Pre (N = 214)	Post (N = 210)	Pre (N = 217)	Post (N = 210)	Pre (N = 644)	Post (N = 630)
Don't Know	57%	41.9%	54.5%	38.6%	49.0%	45.2%	54.0%	41.9%
Keeping the environment clean	1.9%	8.1%	0.5%	1.0%	1.4%	4.3%	1.3%	4.4%
Treatment, vaccination and management of sores	38.0%	48.6%	41.1%	76%	44.0%	64.3%	41%	63.0%
Distribution of Mosquito nets	0.5%	15.2%	0.0%	11.4%	0.0%	32.9%	0.2%	19.8%

#### Knowledge and purpose of MDA

Overall 67.2% of the respondents had heard about the drug distribution in their zones in the pre-intervention survey (Table 7). The knowledge of MDA increased to 75.9% in the post-intervention assessment. Similar trends were observed in Ashiedu Keteke and Ayawaso. Regarding the purpose of the drug distribution, 91.5% of respondents agreed that the purpose of the MDA was to prevent elephantiasis in the pre-intervention survey (Table 8). However, less than one percent indicated “the prevention of Hydrocele” as one of the goals of the drug distribution. On the question of whether public education was done before the MDA, only 29.8% of respondents indicated that there had been public education prior to the mass distribution exercise in the pre-intervention survey (Table 9). This increased to 38.3% in the post-intervention survey. Respondents in the pre-intervention assessment identified ten issues concerning MDA that they would like to know about. The majority simply wanted to be educated about the distribution process and objectives of the MDA. The issues identified by the respondents, on which education should be based included: the basic cause and prevention of the disease, drug effectiveness and safety, education about the drug distribution, source of the drug, sustainability of the exercise, when the distribution will be done again, who should take the drug, reasons for taking anthropometric measurement before medication is given, why the distribution is not done routinely at health facilities and the reasons why the distribution is done annually.

**Table 7:** Knowledge on mass drug distribution by sub metro.

Sub Metro	Ashiedu Keteke		Ledzokuku		Ayawaso (Control)		Overall	
Intervention	Pre (N = 213)	Post (N = 210)	Pre (N = 214)	Post (N = 210)	Pre (N = 217)	Post (N = 210)	Pre (N = 644)	Post (N = 630)
**Yes**	71.8%	83.3%	69.2%	68.9%	60.8%	75.5%	67.2%	75.9%
**No**	28.2%	16.7%	30.8%	31.1%	39.2%	24.5%	32.8%	24.1%

**Table 8:** Purpose of the drug distribution in the pre-intervention survey.

	Ashiedu Keteke	Ledzokuku	Ayawaso (Control)	TOTAL
**Prevent/Treat Elephantiasis**	92.8%	91.9%	89.5%	91.5%
**Don't Know**	7.2%	5.4%	8.3%	6.9%
**Prevent/Treat Hydrocle**	0.0%	2.0%	0.8%	0.9%
**For our health**	0.0%	0.0%	0.8%	0.2%
**To prevent worms**	0.0%	0.0%	0.8%	0.2%
**Worms treatment**	0.0%	0.7%	0.0%	0.2%

**Table 9:** Public education before distribution.

Sub Metro	Ashiedu Keteke		Ledzokuku		Ayawaso (Control)		Overall	
Intervention	Pre (N = 213)	Post (N = 210)	Pre (N = 214)	Post (N = 210)	Pre (N = 217)	Post (N = 210)	Pre (N = 644)	Post (N = 630)
**Don't Know**	7.8%	6.9%	9.5%	18.1%	15.2%	34.6%	10.6%	11.9%
**No**	59.5%	49.7%	69.6%	45.8%	48.5%	53.5%	59.6%	49.8%
**Yes**	32.7%	43.4%	20.9%	36.1%	36.4%	34.6%	29.8%	38.3%

Respondents in the pre-intervention survey indicated three main sources of information about MDA namely health workers (28.3%), radio (20.9%) and community volunteers (20.4%). Posters (1%) and church/mosque (1%) were the least used methods for informing the community about the MDA. During the intervention phase of the study, information vans were used and this led to information vans (36.7%) being the preferred means of information, followed by health worker (24.3%) and radio (20.6%), in the post-intervention assessment (Table 10). It is however worth noting that the use of mobile vans in Ayawaso was not as part of the intervention, but used solely at the discretion of the health workers in the sub-metro area. A follow up question on other ways to provide information to the community indicated that 60% to 64.1% of the people surveyed would prefer to receive information through the media (specifically the radio).

**Table 10:** Sources of information on MDA.

Sub Metro	Ashiedu Keteke		Ledzokuku		Ayawaso (Control)		Overall	
Intervention	Pre (N = 213)	Post (N = 210)	Pre (N = 214)	Post (N = 210)	Pre (N = 217)	Post (N = 210)	Pre (N = 644)	Post (N = 630)
**Radio**	19.0%	8.6%	21.4%	24.8%	21.8%	28.6%	20.9%	20.6%
**Television**	5.6%	4.3%	9.9%	3.8%	7.6%	8.6%	7.9%	5.6%
**Health Worker**	31.7%	13.8%	28.0%	10.0%	25.9%	49.0%	28.3%	24.3%
**Posters**	0.7%	4.3%	1.1%	0.00%	1.2%	0.5%	1.0%	1.6%
**Family Members**	4.9%	5.2%	2.2%	1.4%	2.9%	14.8%	3.2%	7.1%
**Church/Mosque**	0.7%	1.4%	0.0%	2.9%	2.4%	0.5%	1.0%	1.6%
**Community Volunteers**	22.5%	7.6%	18.1%	18.1%	21.2%	6.2%	20.4%	10.6%
**Gong gong**	2.1%	0.5%	1.6%	3.3%	2.9%	1.0%	2.2%	1.6%
**Neighbours/Friends**	12.7%	16.7%	17.6%	7.1%	14.1%	21.4%	15.0%	15.1%
**Information van**	-	65.7%	-	22.4%	-	21.9%	-	36.7%

Drugs were distributed mainly by moving from house-to-house, or central point distribution such as schools, markets and community centers. The house to house distribution was the preferred method in both the pre-intervention (71.0%) and the post-intervention (71.7%) surveys (Table 11). In terms of evaluating the drug distribution coverage, there was an average of 4 persons in a household with a range of 1–21. In Ayawaso where no interventions were implemented reported coverage declined from 67.2% to 58.4%, while in Ledzokoku Krowor which is also one of the intervention sub-metropolitan districts, reported coverage again declined from 82.5% in the pre-intervention phase to 61.6% in the post-intervention phase. However, in Ashiedu Keteke, reported coverage improved from 59.1% in the pre-intervention phase to 86.0% (Table 12) in the post-intervention phase. The surveyed coverages however decreased from the pre-intervention to post-intervention phase, in all areas. The failure of some community members to take the drug can be highlighted by a comment below, by one of the community leaders, during the FDG.

-They will not take the drugs because they do not know why they have to take the drugs and do not know the effect of the drugs on them. If it is explained to them well they will take the drugs and also pass the information on to others (FGD Community leaders).

**Table 11:** Distribution of drugs during MDA.

Sub Metro	Ashiedu Keteke		Ledzokuku		Ayawaso (Control)		Overall	
Intervention	Pre (N = 213)	Post (N = 210)	Pre (N = 214)	Post (N = 210)	Pre (N = 217)	Post (N = 210)	Pre (N = 644)	Post (N = 630)
Church/Mosque	0.5%	0.0%	0.5%	0.0%	1.9%	0.0%	0.9%	0.0%
Community Centre	6.3%	5.7%	7.0%	3.3%	6.9%	15.7%	6.7%	8.3%
Home to Home	71.0%	78.6%	73.0%	64.3%	68.0%	72.4%	71.0%	71.7%
Market	9.0%	16.7%	3.8%	1.4%	9.4%	6.7%	7.3%	8.3%
Schools	11.0%	11.0%	14%	4.8%	10.0%	21.0%	12.0%	12.2%
Chief Palace	0.0%	0.5%	0.0%	0.5%	0.0%	0.0%	0.0%	0.3%

**Table 12:** Treatment coverage.

Metropolitan District/Sub-district	Pre-intervention Coverage		Post-intervention Coverage	
	Reported	Surveyed	Reported	Surveyed
**Ashiedu Keteke**	59.1%	50.2%	86.0%	41.3%
**Ledzokoku**	82.5%	49.1%	61.6%	28.0%
**Ayawaso (Control)**	67.2%	38.2%	58.4%	32.8%

During the pre-intervention survey, more than half of respondents 349/644 (54.2%) reported not taking the drugs during the last distribution. For those who took the drugs, the reasons for taking them included: the prevention of disease, the involvement of government, that drugs were free, that they had taken drugs in in previous years, the absence of side effects in previous MDAs, and being present during the MDA. Reasons provided for the refusal to take the drugs included: side effects of the drugs, religious reasons and not having the disease (Table 13). The main reason for not taking the drug was the fear of the side effects (40%), while another 40% simply had no reasons. 0.2% were absent at the time of the distribution. There was a marginal improvement in these responses during the post-intervention survey. The fear of side effects was identified in the comment below, by one of the community leaders, during the FDG.

-People said they took the drugs and their bodies got swollen so they will not take it again. Some people said when they take it they scratch their bodies a lot (FGD Community leaders).

**Table 13:** Reasons provided for not taking the drugs.

Sub Metro	Ashiedu Keteke		Ledzokuku		Ayawaso (Control)		Overall	
Intervention	Pre (N = 213)	Post (N = 210)	Pre (N = 214)	Post (N = 210)	Pre (N = 217)	Post (N = 210)	Pre (N = 644)	Post (N = 630)
Fear of side effect	40.9%	47.1%	35.1%	19.0%	46.0%	49.0%	40.0%	38.4%
Don't Know	39.0%	28.6%	45.0%	41.9%	35.0%	14.8%	40.0%	28.4%
Don't Understand the reason for the distribution	5.8%	9.5%	7.9%	8.1%	10.0%	20.0%	7.9%	12.5%
Takes alcohol	7.1%		0.0%		0.8%		2.8%	
Don't believe in the drug, Prefer local medicine	2.6%	1.4%	2.6%	0.5%	2.4%	4.3%	2.5%	2.1%
Believe can't get the disease	1.3%	6.2%	4.6%	2.9%	0.8%	8.1%	2.3%	5.7%
Can't take un prescribed drugs from unknown people	0.6%		0.0%		3.9%		1.4%	
Don't like medicine	1.3%	2.4%	2.0%	0.5%	0.0%	1.0%	1.2%	1.3%
Expired Drugs	1.3%		2.0%		0.0%		1.2%	
Had not eaten	0.0%		0.7%		0.0%		0.2%	
Too old to take the drug	0.0%		0.0%		0.8%		0.2%	
Absent	0.0%		0.0%		0.8%		0.2%	

The awareness of side effects increased from the pre-intervention assessment (25.7%) to the post-intervention survey (29.6%), Table 14. Respondents in the pre-intervention survey mentioned nine different side effects with rashes (26.6%), itching (24.9%) and fever (12.4%) being the most mentioned side effects. Other side effects mentioned were: body soreness, paralysis, death, diarrhea, dizziness, weakness and stomach pain, generalized weakness and faster heartbeat (Table 15). In the post-intervention survey, stomach ache, diarrhea and dizziness were also identified.

**Table 14:** Awareness of side effects.

Sub Metro	Ashiedu Keteke		Ledzokuku		Ayawaso (Control)		Overall	
Intervention	Pre (N = 213)	Post (N = 210)	Pre (N = 214)	Post (N = 210)	Pre (N = 217)	Post (N = 210)	Pre (N = 644)	Post (N = 630)
**Don't Know**	1.3%	6.3%	4.8%	9.7%	11.4%	1.3%	5.6%	5.6%
**No**	74.0%	59.1%	68.5%	75.0%	62.9%	61.6%	68.8%	64.7%
**Yes**	24.7%	34.7%	26.7%	15.3%	25.8%	37.1%	25.7%	29.6%

**Table 15:** Side effects identified by respondents in the pre-intervention assessment.

Side Effects	Ashiedu Keteke	Ledzokuku	Ayawaso (Control)	TOTAL
Rashes	16.4%	35.3%	26.1%	26.6%
Itching	25.5%	26.5%	21.7%	24.9%
Fever	14.5%	8.8%	15.2%	12.4%
Headache	12.7%	10.3%	13.0%	11.8%
Muscle/Bodily Pain	10.9%	7.4%	8.7%	8.9%
Vomiting	5.5%	1.5%	8.7%	4.7%
Swelling Parts of Body	5.5%	8.8%	2.2%	5.9%
Chills	7.3%	0.0%	0.0%	2.4%
Fainting	1.8%	1.5%	4.3%	2.4%
**TOTAL**	100%	100%	100%	100%

### Provider challenges

Many challenges to MDA were also reported following interviews with various providers and facilities heads. First among the challenges was the inadequate remunerations and motivation. Health providers were interested in knowing how much they will be paid before they attend training for MDA. Remuneration only after the training or work has been done was not appreciated. Below is a statement from one of the FGD members.

- The allowance is not good at all but for us is like it is our work. For the allowance to be honest it is not good at all (FGD Implementer).

Another challenge identified, in all three sub metro areas, was the lack of some logistics to assists in MDA, such as rain coats, vehicles for supervision, and stationery (reporting forms, pen, and pencil). These were reported to be either inadequate or not available. Providers reported that often the program is poorly planned and impromptu arrangements are made, with training done on the spur-of- the moment, which was not ideal. As such, complaints about the delay in the provision of logistics as a result of poor planning are a challenge to drug distribution. Health providers indicated that their exclusion in the planning process for the drug distribution, and their involvement only when their services are needed was not the best and would prefer to be involved at all stages. The sentiment below reflects some of these views.

- We haven’t heard anything. All of a sudden … they will call us and say we are doing this training tomorrow. Everything is done in a day and then they say we should come and implement it (FGD Provider).

The supervisors also reported that the dress code of some distributors was unacceptable to some community members and that may contribute to refusal of some people to accept volunteers in their homes and take the drugs. As such, they suggested T-shirts for volunteers rather than allowing them to wear their own clothes. Some of the supervisors were of the view that volunteers needed to be provided with some form of identification cards to ensure their acceptability and trust by community members.

Even though the supervisors have been working with the drug distributors over the years there was suspicion that drug distributors throw away drugs when they are unable to distribute them, due to the large coverage targets they are to meet. Reasons for throwing away these drug are not clear. Providers however indicated that they encourage distributors to desist from such acts. These sentiments are captured in the sentence below.

- … they throw the medicine away and come back. That is why I tell them that when you go and don’t meet anyone there, you bring the medicine back. Don’t throw it away. God is looking at you. You are just spoiling the medicine because you feel it is free but they bought it. (FGD provider).

One of the main concerns of providers and distributors in some of the communities is the consumption of alcohol by community members making it difficult for distributors to give them drugs to take during MDA. Other impediments have to do with food and water. Some community members ask that they are provided with food and water or money to buy them when they have not taken in any food at the time of arrival of drug distributors.

Finally, the providers reported that early awareness creation prior to the distribution of drugs is important for effective distribution and acceptability. Both health care providers and volunteers stressed the importance of creating awareness and public education and information using the mass media and other available communication strategies as key to a successful implementation of MDA.

### Volunteer challenges

The volunteers also described their challenges with respect to the drug distribution. Remuneration was the main concern for volunteers. Volunteer allowances were considered insignificant. Volunteers explained that they experience difficulties in reaching communities of higher socio-economic status. Convincing them to take drugs was a challenge among community members. The feeling of disrespect to CDDs by residents in these communities was another challenge. Volunteers felt intimidated when they were not able to express themselves well in English and were not able to respond to questions posed by these residents adequately. In some of these communities school authorities asked for parental consent before any child was given the drug. This then means that school authorities need to be informed prior to any drug distribution. Without parental consent a child will not be allowed by school authorities to take the drug. Even with parental consent school authorities are reluctant to allow children from high-income communities to take the drug. Also, some community members want to be sure distributors are truly sent by the government to distribute drugs. Some families and school heads ask for introductory letters from distributors.

Volunteers suggested that the program provide T-shirts or other form of identification when they visit such communities. Many people in the urban communities insist on knowing whether the volunteers are genuine before allowing drug distributors to give drugs to their households.

Some concerns of the distributors are reflected in the comments below:

- The allowance is not good at all but for us it is like it is our work. For the allowance, to be honest, it is not good at all (FGD Implementer).

-When you are not a nurse, they won’t even look at you. There are schools, when you are going for immunization and you send volunteers there they will not allow volunteers to do it unless you go yourself as a nurse.

-We need Identity cards or even T-shirts with an inscription that will specify the work we do. A woman didn’t allow us to give the drug to her child because nothing shows we are genuine. (Female Distributor).

- Please another problem is that, when we go for training knowing that this is what we are going to do, they should let us know, tell us the allowance they are going to give you. Because at times you finish the work and they will give you what they want (FGD Implementer).

### Intervention phase

Following the pre-intervention assessments, several recommendations were made with regard to improving MDA implementation in urban areas. Based on recommendations made by both health workers and community members interviewed during the pre-intervention survey, meetings were held with health workers at the regional, metropolitan/ and sub-metropolitan level. Many recommendations were made following the pre-intervention phase. It was operationally impossible to implement them all and therefore after discussions with the implementing districts, some of them were selected for implementation. Table 16 summarizes the issues identified, the solutions and interventions that were implemented. The study team and the national NTD program office provided direct support and guidance to the implementing districts at all stages of implementation. While the interventions were specifically implemented in Ashiedu Keteke and Ledzokuku, the study team made no input into the activities undertaken in Ayawaso. Any activity undertaken in Ayawaso, such as the use of mobile vans for social mobilization, was carried out at the discretion of the health workers.

**Table 16:** Interventions for improving urban drug delivery.

ISSUES/ CONCERNS	CHALLENGES	SOLUTION	INTERVENTION
Community definition	• Communities not well defined for the exercise. • Zones are rather used as the smallest operational unit	• Communities or operational units to be redefined and listed • Communities should be dealt with independently	• Define and list communities or operational units of all zones • Treat each community as an independent entity
Selection of CDDs	• Temporary volunteers selected to do work • CDDs cannot be followed up for drugs or report side reactions • Low commitment of CDDs • CDDs cannot be retained for subsequent MDAs	• Selected CDDs must be available to do the distribution/work • CDDs must be resident in the community • CDDs must volunteer to serve • Ensure training for CDDs every year	Select people, with the involvement with community: • Available to do work always • Resident and known in the community • Who demonstrate interest and commitment
Training of CDDs	• Inadequate training of CDDs to respond to community issues on the MDA • CDDs unable to explain the importance of the exercise well and the need for all to participate	• CDDs must be able to explain the exercise well to build trust and encourage participation • CDDs must be able to convince people to participate in the exercise	• Training of CDDs detailed to equip them well for the task. However, this was basic enough and the period limited to less than a day, so as to enhance their active participation in the training. • CDDs taught the skills of communication and interaction and the need to be patient and tolerant to even difficult people
CDD identity	Inadequate/lack of identification documents	Provide CDDs with: • Introductory letters • ID cards • T-shirts for CDDs	• Letters sent to institutions on MDA • Use available ID cards • Use branded T-shirts (if available)
Volunteer motivation or incentive	Inadequate remuneration and complaint by CDDs of lack of transparency in the payments	Enhance communication with CDDs to improve transparency regarding the remuneration. Improve the remuneration package	• Volunteers made to know, from the beginning, how much is due them for the exercise • Volunteers paid exactly how much they are promised
Social Mobilization	Little or inadequate information prior to treatment	Use the media for social mobilization: • Radio • TV • Mobile van announcements • Churches/Mosques • Social/ Professional groups	Social mobilization done using the following media: • Radio • TV • Mobile van announcements • Churches/Mosques Social/ Professional groups e.g. Meetings with Tailors & Seamstresses Associations, etc.
Drug distribution Process	• Treatment not able to reach people in: ○ Markets ○ Institutions and offices High income residential areas • CDDs don’t feel comfortable working in middle class areas • Duration of exercise not enough to cover most people • Treatment done in a sweeping form where many CDDs are used to work in a community in a day, finish and move to the next • Exercise carried out at times when there are other competing health programmes, making it difficult for health workers to devote the needed time and attention to the exercise • Inadequate time for preparation	• Reach people in markets, institutions, offices and first class residential areas with treatment • Sending people with higher qualification and good knowledge of the disease and MDA to these areas to treat • Enough days given for the exercise • CDDs assigned to respective communities to do the work on those communities through the whole duration of the exercise • The exercise be timed not to coincide with any other competing programmes • Explore integration or co-implementation with other programmes • The exercise is done in a rush such that not adequate time is allowed for preparation and enough social mobilization	Distributors sent to markets, institutions, offices and high income residential areas (with ID cards), after adequate social mobilization • Health workers in their uniforms, who are able to explain the disease and the MDA, sent to distribute the drugs to people living in middle class areas who might intimidate the CDDs. • Enough days given for the exercise and also give room for mopping up when need be • CDDs assigned to defined communities which they will be responsible for throughout the whole duration of the exercise • The exercise timed not to coincide with any other competing programmes • Preparations for the exercise started in good time to allow for enough preparation and enough social mobilization prior to the MDA
Adverse Reactions	• Community members do not know what to do when they experience adverse reactions • People are not aware of available help in case of adverse reactions • Community members do not know who to report adverse reactions to	• Provide information on what people should do in the event of adverse reactions • Information on interventions made available to people • Information on the chain of reporting of adverse reactions must be provided	• As part of the social mobilization, people were made to know what possible adverse reactions there are to the drugs and what to do when they occur • People informed that there is free care in the event of reaction when reported and the channels to be followed • People were informed, during social mobilization, to report cases of adverse reactions to the CDDs or nearest facility indicated
Feedback to communities	• No feedback is given to communities on the MDA and the status of the disease prevalence of the community or district	• Give feedback on the MDA and the status of the disease in the district	• Through the CDDs or Zonal staff, feedback was given to the communities

The findings of the pre-intervention survey were disseminated at the regional review and training of trainers for the 2012 MDA. Meetings were also held with the District Health Management Teams (DHMTs) to further discuss the details of the findings with them to ensure better understanding of the issues. Many meetings were held with the DHMTs to discuss and agree on which recommendations to implement and what is involved in carrying them out. The details of the issues to address and the overall scope of work were examined. The need to change some of the ways of routinely implementing the program was stressed.

#### Planning and budgeting

The national program team participated in district MDA planning meetings for the implementing districts. A series of these meetings were held during which the districts discussed the recommendations from the study, which of them were implementable within various constraints, what it would take to implement them and how to execute the exercise.

The available budget for the routine MDA was looked at vis-à-vis the scope of the work and where the need for additional funding arose, it was discussed and supplementary budget raised to meet the additional work required in the two intervention districts.

#### Training activities

The national team was involved in the training of municipal, sub-metro, and zonal supervisors, CDDs and their supervisors. They were taken through the objectives of the implementation of the recommendations and the roles and responsibilities. Also addressed were the new and additional ways of conducting the MDA.

#### Social mobilization

Social mobilization for MDA implementation, started just after the regional level training of trainers. It took the forms of TV and radio discussions, community meetings (where the purpose of the MDA was explained to community members in order to seek their active participation and support), meetings with community leaders (chiefs and elders, and assembly members), meetings with the local government authorities (metropolitan and municipal assemblies), forum with institutions and recognizable groups, mobile van announcements, one-on-one education by health workers and CDDs. Materials used for education and social mobilization included flyers and a documentary on NTDs.

#### MDA implementation

The MDA followed the routine strategies, with some modifications. The challenges to MDA presented in Table 16, were addressed and implemented during the intervention. Routinely, treatment was done from house to house, schools were visited for treatment. The treatment was not able to reach people in: markets, institutions and offices, high income residential areas. CDDs did not feel comfortable working in high income areas and the duration of the exercise was not enough to cover most people. Also, treatment in communities was done in a sweeping form where many CDDs were used to work in a community in a day, finish and move to the next. The exercise was carried out at times when there were other competing health programs, making it difficult for health workers to devote the needed time and attention to the exercise.

However, for the intervention, a mobile team moved with a van announcing the treatment and also treating people who came to the van. Teams visited schools to treat children, after prior notification was sent to the schools. Treatment was also done at the markets and sometimes at churches and mosques. The districts were operationally divided into zones. Work in the districts was monitored and supervised by coordinators, and in the zones it was supervised by zonal heads who were assisted by team supervisors. The team supervisors supervised 5 or 6 teams, each of which was made up of 2 CDDs. Communities were demarcated into sections and marked according to days they were to be visited. Teams under a supervisor worked in marked sections in a “sweeping” fashion, one after the other (i.e. the whole group moved to a section, finished the work there and moved to the next). The last day was used to mop up through all the sections of the area the group had been allocated. Communities were not assigned specific permanent CDDs to work in throughout the treatment period and subsequent exercises.

The basic unit for treatment and reporting in the urban areas (mainly in the Greater Accra region) was the zones instead of communities as is the case of rural districts. The CDDs did not have to reside in the communities they worked in since the teams finished with a section and moved to the next till they covered the area within the treatment period.

The following additional strategies were implemented.

- Identification and listing of all communities in the districts
- Assignment of specific CDDs to communities which they would be responsible for treating throughout the period and subsequently
- CDDs assigned to communities were residents of those communities, as much as possible. In high income areas, community health nurses were engaged to administer the drugs in communities or areas that the elites reside to increase acceptance
- The districts health workers held meetings with community leaders for their support for the exercise
- Meetings were held with the local government authorities to elicit support
- Educational fora were organized in institutions and organizations within the districts to enhance participation
- Community durbars were organized to ensure more understanding and participation by community members
- The number of CDDs was increased to reduce workload and improve coverage. The implementing sub-metros were given 25% additional CDDs (from 200–250 CDDs).
- The remuneration of CDDs was increased (though marginally) to increase their enthusiasm and commitment. About 20% additional funding (10,000–12,000 GHS) was given to support the activities.
- Drug distribution period was extended (from 3 days to 1 week)
- Different drug distribution strategies and timing were adopted for different communities informed by their peculiar issues
- Drug distribution points were created outside churches and mosques for members to take the drugs after service
- Drug distributors visited markets to distribute drugs

#### Key implementation challenges in intervention areas

Several challenges were encountered during the intervention phase, and are briefly described below. Many competing programs put pressure on health workers and CDDs. The MDA immediately followed either the National Immunization Day (NID) campaign. As such, the CDDs went into the MDA already exhausted and less motivated to do the work well. Further, the low remuneration for CDDs affected their recruitment, especially in Ledzokuku Krowor municipal where many of the old CDDs refused to take part. This became a bigger problem as the MDA came just on the heels of the NID where people had to do less work and received higher remuneration, thus making participation in the MDA activities less attractive. This issue remains a major challenge to urban drug distribution and the success of the work largely depends on solving this challenge.

There were difficulties in getting resident CDDs to distribute the drugs in their communities, thus making the MDA more difficult and expensive. As a result, CDDs were recruited from other communities and transported to and fro on daily basis. Given the timing of MDAs, there was also inadequate time for sufficient social mobilization prior to the drug distribution. The best time to implement MDA activities would be from January to March. However, funding support for MDA activities is usually delayed, leading to inadequate timing for social mobilization. As a result, MDA happen in June/July or later on in the year.

The research team observed other challenges in the implementation of the interventions, and it is essential to address these in order to successfully address coverage in urban areas. These challenges include poor leadership, low commitment of health workers to implement the recommended interventions, lack of proper planning by the health workers and little involvement of the community members.

The biggest challenge that affected the implementation of the recommendations of the study was funding. This greatly affected the ability to pay realistic remuneration to drug distributors and this caused the refusal of many CDDs to participate in the MDA activities. The program also had challenges providing T-shirts for CDDs, for easy identification and motivation.

## Discussion

This study was undertaken with the aim of improving the implementation of MDA in urban areas, through the identification of opportunities and barriers for implementation. In this study, interventions were undertaken in Ashiedu Keteke and Ledzokuku, while Ayawaso was considered a control site with no intervention. Overall, the results indicated that where interventions were undertaken, there was an improvement in the knowledge, attitude and practices of respondents. However, the interventions did not have the desired effects on coverage rates. In Ashiedu Kekete, where the research team observed the zeal and commitment of the leadership to improve on the coverage during the intervention phase of the study, the interventions implemented played a role in the observed improvement in reported coverage, even though the surveyed coverage declined. This commitment was demonstrated during the planning process of the intervention, where the district director and disease control officer readily worked to produce an implementation plan and budget based on the selected recommendations for implementing the interventions. They readily accepted to work with the limited funding provided for the interventions. In the case of Ledzokoku Krowor, this commitment was totally missing. Much of the planning was done by the research and program team on behalf of this district. They demanded more funding for implementation, which was not available. They also ended up implementing just a few of the selected recommendations during the intervention phase. Ayawaso being a control district did not implement any of the interventions but was only routinely followed up as one of the program districts and here the reported coverage declined from the pre-intervention phase to the post-intervention phase. These observations reveal the complexity of working in urban areas, and that health workers understand their system and difficulties at the ground much better than the national level team. In the process of this study, it became much clearer to the NTD programme that leadership, funding, planning and community involvement are very important in the success of MDA campaigns in urban areas.

The evaluation revealed a substantial lack of knowledge, low risk perceptions and understanding of LF and its control by study respondents in the urban areas surveyed. Similar observations have been made elsewhere [13,14], and reflect the need for the creation of awareness, health education (through the media) and involvement of the urban communities and individuals in deciding on the materials and methods to be used in LF control activities [15,16]. The importance of socio-economic status proved to be a problem in the implementation of urban MDA, similar to other studies [13,17]. Motivation of CDDs was also an extremely important factor that determines the success of MDA in urban areas [18]. In addition to the financial concerns raised by the CDDs, their numerical strength and the length of the drug distribution period were other issues that need to be addressed in improving MDA in urban areas. These concerns seem not to be limited to Ghana only, but other countries as well [15,18].

The dissemination of information in order to enhance the public acceptability of public health interventions is very critical. In this study, majority of respondents indicated that they would prefer to receive information through the media (specifically the radio). The media plays a significant role in the formation of public opinion, especially where public knowledge is low [19]. In Ghana, there is a nationwide radio coverage, with up to 69% of households owning a radio [20]. In urban areas, the coverage is even higher with 72.5% of urban populations owning a radio set. This makes the media a very important source of information whereby households can receive updated daily news events, information, and educational materials. Majority of radio stations in Ghana provide information in local languages, and this makes radios convenient at night when health workers are not around to give out the information. Public education campaigns prior to MDA in urban areas can therefore capitalize on this in order to disseminate information to urban populations.

The nature, composition and dynamics of urban populations may require that MDA activities are tailored to particular groups [11,15]. The use of education materials should focus on the local environment, health system, social structure, culture, population density and method of drug distribution [21]. This study also revealed that the knowledge of the disease was generally low. Where knowledge and risk perceptions are low, people give low priority to disease prevention [22]. However, the health education provided in Ashiedu Keteke therefore led to an improvement in the knowledge, attitude and practices relating to the disease, and an increased risk perception regarding elephantiasis (the symptom). The fear of the side effects associated with the drug was an important factor that influenced the decision to take the MDA drugs and has been identified in several studies [23,24]. In this study, the fear of the adverse reactions improved from the pre-intervention to the post-intervention phase, indicating that this can be overcome following appropriate education on possible adverse reactions, the reasons for their occurrence–in this case a sign that the drugs are killing the parasites and getting rid of the infection, and how to manage them. While a number of administrative challenges to MDA in urban areas were also identified, proper mobilization of resources and advocacy may help overcome these challenges associated with the planning of MDA [9] in urban areas.

This study had a number of limitations. First, a higher number of female respondents were interviewed, with the likelihood of biasing the outcomes of the survey. The high number of female respondents could be explained by the fact that the males were the major bread winners, especially in the low income communities, leaving the women to take care of the house and the children. As such during the survey, most men were out of the house. Another challenge in this study, was the lack of disaggregation of the data to look at socio-economic status and KAP responses of study respondents. As a results, there was the failure to undertake correlation analyses to determine the impact of socio-economic status on the risk perception of the disease. The demographic characteristics of the participants in the FDGs and IDIs were also not recorded. A further challenge presented by this study was the low surveyed coverage observed. Undertaking coverage surveys during weekdays posed the challenge of locating individuals who have been treated during the MDA implementation in these highly populated urban areas, bustling with economic activities. As will be observed, surveyed coverage decreased for both the intervention and control sub-metropolitan districts. The use of reported coverage versus surveyed coverage in urban areas faces interpretation challenges. The reported coverage has challenges with the denominator (estimated population based on the latest census records). In urban areas, unlike rural areas there are no community registers and tally sheets are used to note whoever was available and treated at the time of the MDA. Therefore, the best indicator for monitoring MDA in urban areas is the absolute numbers reported since the denominator might not represent the population under discussion. On the other hand the survey coverage has challenges with the actual people surveyed and in urban dynamic areas where people are constantly moving in and out (looking for better accommodation, moving closer to workplaces, etc…), it is actually difficult to find the same people, compared to rural areas. Urban populations are very mobile and unpredictable especially among urban slums and indigenous urban communities. In these areas where MDAs take place in areas where the population aggregate during the day such as markets, schools and work places, most of the people treated are not residents of these areas and therefore not traceable, leading to discrepancy between the reported and surveyed coverage. The time lapse between the treatment and the survey further compounds the challenges in a dynamic urban population, and therefore rapid coverage assessments should ideally be done during or within days of completion of the MDA. Further the cluster method [12] used for the selection of households for the coverage survey maybe more suitable for rural areas than urban areas. Nonetheless current guidelines for implementing coverage surveys [25] may help address these challenges in urban areas. The timing of the MDA and coverage surveys may have also contributed to the low coverages and perhaps undertaking MDAs and surveys on weekends when most individuals will be at home may increase the surveyed coverage observed in urban areas. However, this is not feasible, considering the logistics, time, financial and personnel constraints on programs. Based on these consideration, the reported coverage in Ashiedu Keteke, where interventions were readily implemented, maybe considered to be a much higher improvement. Finally, the survey showed that the populations pre and post intervention were different, and may not be representative of the entire population, and this may explain the differences in the responses obtained. However, surveying different pre and post populations may actually be beneficial as this may reflect the dearth of the public education campaigns.

This study provides an avenue for better understanding the challenges to MDA implementation in urban areas, with far reaching consequences for MDA implementation in all other districts. For example, studies assessing MDA compliance rates in the Ahanta West district of Ghana [26] revealed that compliance was significantly lower (43.8%) than that reported by the community-based volunteers (83.6%), and the odds of not receiving the drugs were significantly associated with side effects, and low risk perception. The study further emphasized the need for improved health education focusing on the safety of drugs and the importance of MDA before and during the drug distribution exercises. Another study evaluating the quality of data reported from some of the districts in Ghana revealed inaccuracies and exposed challenges and limitations of the data management system [27]. In the study presented herein, a host of issues have been identified that will need to be individually examined, and addressed if MDA in urban areas is to be improved. These include (but are not limited to) administrative, health systems, leadership, financial and community participation, and form the basis on which smaller studies can be undertaken. The concerns identified by providers and volunteers represent administrative and health systems challenges that could be tackled through quality improvement activities. Together, these examples point to a substantial challenge with MDA coverage in Ghana, and suggest that coverage needs to be improved by strengthening the operational processes, management role, conduct of MDA, effectiveness of reporting systems in order to achieve the 2020 implementation goals. As such, there is the need for quality improvement processes [28–30], for MDA in urban and other areas, aimed at improving the efficiency and processes of the program, through systematic and continuous actions that lead to measurable improvement in the provision of health care services.

### Conclusions

The observations from this study showed that, generally knowledge, attitudes and practices of community members to MDA improved slightly from the pre-intervention phase to the post-intervention phase. However, the intervention did not result in an increase in the number of people receiving the drug in all districts. Many factors were identified as being important in improving implementation and improving coverage of MDA in urban areas. Significant among these are leadership, planning, funding, developing an ideal work force of both health workers and community drug distributors, involvement of community members and knowledge of the disease targeted by the MDA drugs. Implementing MDA in urban areas therefore needs to be given significant consideration and planning, taking into consideration quality improvement processes, if the aims of achieving the required coverage rates are to be achieved.

## Supporting information

S1 File Focus group discussion guide. (DOCX)

S2 File In-depth interview guide. (DOCX)

S3 File Household survey questionnaire. (DOC)
