# Supplementary material for: Improving drug delivery strategies for lymphatic filariasis elimination in urban areas in Ghana
Source: PLoS Negl Trop Dis. 2017 May 11;11(5):e0005619. doi: 10.1371/journal.pntd.0005619 (PMC5441634; doi:10.1371/journal.pntd.0005619)
Supplement: S1 File — (DOCX) [file pntd.0005619.s001.docx]

**S1 FILE: ALTERNATIVE DRUG DELIVERY STRATEGIES FOR LYMPHATIC FILARIASIS ELIMINATION IN URBAN AREAS IN GHANA**

**FOCUS GROUP DISCUSSION GUIDES**

***Introduction***

*My name is …………………..and I work for the Ghana Health Service. We are conducting a study to gather information on Mass rug Distribution with Ivermectin and Albendazole programme in selected urban areas in Greater Accra Region. Note that I am referring to the distribution of the two drugs for which the heights of the people are measured before the drugs are given.*

*The purpose of this study is to gather information that will help us to know what happened with previous mass drug treatment exercise and to learn lessons that will help us to improve on future mass drug distribution exercises in urban areas such as yours.*

*We will try not to interrupt when you are speaking and also to allow you to say all you want to say. To capture all this information well, we would like to use a tape recorder during the discussion, however I assure you that all the information gathered will be put together at the end and what you say cannot be linked to you. The tape will be destroyed after we have taken all the information from it.*

*We are very interested in your opinions; everything you say is very interesting for us. I don’t want* *to talk much; I want you to talk freely as much as you want. There are no ‘correct’ or ‘wrong’ answers****.***

*Please feel free to express your opinion on the issues to be discussed.*

**FGDs with community leaders (assemblymen, opinion leaders)**

1. What is the demarcation of the community?
2. What is the population of the community?
3. Which are the different ethnic groups?
4. What are the different social and economic groups?
5. What are the different economic activities in this community?
6. What are the different social activities in this community? (Please describe: when; how often; by which people; how can we contact those people)
7. What health related programmes do you have in this community (by whom; on what; for who)
8. What are the other things about this community that you can tell us about?

**Thank you for your time**

**ALTERNATIVE DRUG DELIVERY STRATEGIES FOR LYMPHATIC FILARIASIS ELIMINATION IN URBAN AREAS IN GHANA**

**FGDs with community members (from the different strata)**

***Introduction***

*My name is …………………..and I work for the Ghana Health Service. We are conducting a study to gather information on Mass rug Distribution with Ivermectin and Albendazole programme in selected urban areas in Greater Accra Region. Note that I am referring to the distribution of the two drugs for which the heights of the people are measured before the drugs are given.*

*The purpose of this study is to gather information that will help us to know what happened with previous mass drug treatment exercise and to learn lessons that will help us to improve on future mass drug distribution exercises in urban areas such as yours.*

*We will try not to interrupt when you are speaking and also to allow you to say all you want to say. To capture all this information well, we would like to use a tape recorder during the discussion, however I assure you that all the information gathered will be put together at the end and what you say cannot be linked to you. The tape will be destroyed after we have taken all the information from it.*

*We are very interested in your opinions; everything you say is very interesting for us. I don’t want* *to talk much; I want you to talk freely as much as you want. There are no ‘correct’ or ‘wrong’ answers****.***

*Please feel free to express your opinion on the issues to be discussed.*

1. What do you know about the LF?
2. How is the disease acquired?
3. How can it be prevented?
4. Do you know about the MDA that happens in this community?
5. What do you know about it?
6. How did you know about the drug distribution?
7. What do you think about the process of drug distribution?
8. What do you like about it and why?
9. What do you not like about it and why?
10. What suggestions do you have for the distribution of drugs so many people will take the drug?
11. How can we improve how the drug distribution process?
12. How can we improve the MDA in general?

**Thank you for your time**

**ALTERNATIVE DRUG DELIVERY STRATEGIES FOR LYMPHATIC FILARIASIS ELIMINATION IN URBAN AREAS IN GHANA**

**FGDs with CDDs**

***Introduction***

*My name is …………………..and I work for the Ghana Health Service. We are conducting a study to gather information on Mass rug Distribution with Ivermectin and Albendazole programme in selected urban areas in Greater Accra Region. Note that I am referring to the distribution of the two drugs for which the heights of the people are measured before the drugs are given.*

*The purpose of this study is to gather information that will help us to know what happened with previous mass drug treatment exercise and to learn lessons that will help us to improve on future mass drug distribution exercises in urban areas such as yours.*

*We will try not to interrupt when you are speaking and also to allow you to say all you want to say. To capture all this information well, we would like to use a tape recorder during the discussion, however I assure you that all the information gathered will be put together at the end and what you say cannot be linked to you. The tape will be destroyed after we have taken all the information from it.*

*We are very interested in your opinions; everything you say is very interesting for us. I don’t want* *to talk much; I want you to talk freely as much as you want. There are no ‘correct’ or ‘wrong’ answers****.***

*Please feel free to express your opinion on the issues to be discussed.*

1. How were you selected?
2. What is your work about?
3. Why do you do the work?
4. How do you carry out the work? (procedure)
5. What is your motivation for doing the work?
6. What are the challenges involved?
7. How do the people want you to do the work?
8. What should be done to make your work less difficult?
9. What suggestions do you have so we can reach more people in your community?

**Thank you for your time**
