# Supplementary material for: Improving drug delivery strategies for lymphatic filariasis elimination in urban areas in Ghana
Source: PLoS Negl Trop Dis. 2017 May 11;11(5):e0005619. doi: 10.1371/journal.pntd.0005619 (PMC5441634; doi:10.1371/journal.pntd.0005619)
Supplement: S2 File — (DOCX) [file pntd.0005619.s002.docx]

**S2 FILE: ALTERNATIVE DRUG DELIVERY STRATEGIES FOR LYMPHATIC FILARIASIS ELIMINATION IN URBAN AREAS IN GHANA**

**IDIs for health workers, NGOs and partners and stakeholders**

***Introduction***

*My name is …………………..and I work for the Ghana Health Service. We are conducting a study to gather information on Mass rug Distribution with Ivermectin and Albendazole programme in selected urban areas in Greater Accra Region. Note that I am referring to the distribution of the two drugs for which the heights of the people are measured before the drugs are given.*

*The purpose of this study is to gather information that will help us to know what happened with previous mass drug treatment exercise and to learn lessons that will help us to improve on future mass drug distribution exercises in urban areas such as yours.*

*We will try not to interrupt when you are speaking and also to allow you to say all you want to say. To capture all this information well, we would like to use a tape recorder during the interview, however I assure you that all the information gathered will be put together at the end and what you say cannot be linked to you. The tape will be destroyed after we have taken all the information from it.*

*We are very interested in your opinions; everything you say is very interesting for us. I don’t want* *to talk much; I want you to talk freely as much as you want. Please feel free to express your opinion on the issues to be discussed.*

1. What are the different NGOs and partners that operate in this community? (List all NGOs and Partners that operate in the community)
2. What are their areas of operation?
3. What programmes do they run?
4. Who are beneficiaries of your programmes?
5. How do you reach your targets?
6. How can the NGOs and partners in this area be reached?
7. What challenges do you face in the implementation of your activities?
8. How have you managed these challenges?
9. How do you think we can improve the MDA that we conduct in this community?

**Thank you for your time**

**ALTERNATIVE DRUG DELIVERY STRATEGIES FOR LYMPHATIC FILARIASIS ELIMINATION IN URBAN AREAS IN GHANA**

**IDI Guide with MDA implementers**

***Introduction***

*My name is …………………..and I work for the Ghana Health Service. We are conducting a study to gather information on Mass rug Distribution with Ivermectin and Albendazole programme in selected urban areas in Greater Accra Region. Note that I am referring to the distribution of the two drugs for which the heights of the people are measured before the drugs are given.*

*The purpose of this study is to gather information that will help us to know what happened with previous mass drug treatment exercise and to learn lessons that will help us to improve on future mass drug distribution exercises in urban areas such as yours.*

*We will try not to interrupt when you are speaking and also to allow you to say all you want to say. To capture all this information well, we would like to use a tape recorder during the discussion, however I assure you that all the information gathered will be put together at the end and what you say cannot be linked to you. The tape will be destroyed after we have taken all the information from it.*

*We are very interested in your opinions; everything you say is very interesting for us. I don’t want* *to talk much; I want you to talk freely as much as you want. There are no ‘correct’ or ‘wrong’ answers****.***

*Please feel free to express your opinion on the issues to be discussed.*

1. How are CDDs selected?
2. Are CDDs trained and retrained?
3. What is your work about?
4. Why do you do the work?
5. How do you carry out the work? (procedure)
6. What is your motivation for doing the work?
7. What are the challenges involved in your work as an implementer?
8. How do the people want you to do the work?
9. What should be done to make your work less difficult?
10. What suggestions do you have so we can reach more people in your area?

**Thank you for your time**
