# Supplementary material for: Improving drug delivery strategies for lymphatic filariasis elimination in urban areas in Ghana
Source: PLoS Negl Trop Dis. 2017 May 11;11(5):e0005619. doi: 10.1371/journal.pntd.0005619 (PMC5441634; doi:10.1371/journal.pntd.0005619)
Supplement: S3 File — (DOC) [file pntd.0005619.s003.doc]

**S3 FILE: Alternative Drug Delivery Strategies for Lymphatic Filariasis Elimination In An Urban Area In Ghana**

**QUESTIONNAIRE**

**HOUSEHOLD INTERVIEW**

Name of Interviewer...……………………………………. Date of Interview……………………

Community………………………………………. Suburb or locality…………………………………………

Sub-district…………………………………..District……………………………………………

***Introduction***

*My name is …………………..and I work for the Ghana Health Service. We are conducting a study to gather information on Mass rug Distribution with Ivermectin and Albendazole programme in selected urban areas in Greater Accra Region. Note that I am referring to the distribution of the two drugs for which the heights of the people are measured before the drugs are given.*

*The purpose of this study is to gather information that will help us to know what happened with previous mass drug treatment exercise and to learn lessons that will help us to improve on future mass drug distribution exercises in urban areas such as yours.*

*We will try not to interrupt when you are speaking and also to allow you to say all you want to say. We will only interrupt when you are deviating from the points for discussion.*

*To capture all this information well, we would like to use a tape recorder during the interview, however I assure you that all the information gathered will be put together at the end and what you say cannot be linked to you. The tape will be destroyed after we have taken all the information from it.*

*We are very interested in your opinions; everything you say is very interesting for us. I don’t want to talk much; I want you to talk freely as much as you want. Please feel free to express your opinion on the issues to be discussed****.***

***Interviewer: Ask to speak to all members of the household present who are 18 years and above.***

**Section A: Socio-demographic Data**

**1. Name of respondent …………………………………………………….**

**2. AGE** [ ] [ ]

**3**. **SEX** 1. Male 2. Female

**4. Marital Status** 1. Married 2. Single 3. Divorced 4. Widowed

**5. Number of people in this household [ ] [ ]**

**6. Occupation** 1. Farmer 2. Fisherman 3. Teacher 4. Artisan

5. Trader 7. Other, specify…………………………

**7. Religion:** 1. Christian 2. Moslem 3. Traditional Religion

4.None 7 Other, specify……………………………

**8. Number of completed years of education** [ ] [ ]

**Section B : Knowledge about the Disease**

**9. Have you heard about the disease Elephantiasis?** 1. Yes 2. No

**10. If yes, how does it present in an affected individual? (Multiple correct answers are allowed)**

1. Enlarged Legs 2. Enlarged Scrotum 3. Enlarged Arms 4. Enlarged Breasts

5. Fever and Chills 6. Painful swollen groin

7. Other, specify………………………………………………………..

8. Don’t know 9. N/A

**11. How is this disease acquired? (Multiple correct answers are allowed)**

1. Through the bite of a mosquito 2. Breathing in the germs

3. Eating contaminated food 4. Drinking contaminated water

7. Other, Specify………………………………………………

8. Don’t know 9. N/A

**12. Which germs are responsible for the disease?** **(Multiple correct answers are allowed)**

1. Tiny worms in the body 2. Bacteria 3. Viruses

7. Other, Specify……………………….

8. Don’t Know 9. N/A

**13. Are you are risk of getting this disease?** 1. Yes 2. No 8. Don’t know

**Section C: Management and Programme**

**13. How is lymphatic filariasis disease prevented? (Multiple correct answers are allowed)**

1.By taking drugs 2. Sleeping in Insecticide treated mosquito nets

3. Keeping a clean environment 7. Other, specify……………… 8. Don’t know 9. N/A

14. Are you aware of any drug distribution in this community for which people’s heights were measured? 1. Yes 2. No (If No, skip to Q25)

15. How many different drugs are given during the exercise? [ ] [ ] 99. N/A

16. What categories of people should not take the drugs? (Multiple correct answers are allowed)

1. Children less than a certain height 2. Pregnant women 3. Breast feeding mothers

4. Seriously sick people 7. Others, specify…………………………………

8. Don’t know 9. N/A

17. Did everybody in your household take the drugs?

1. Yes 2. No 8. Don’t Know 9. N/A

18. What reasons do people give for refusing to take the drugs?

…………………………………………………………………………………………….

……………………………………………………………………………………………..

……………………………………………………………………………………………..

19. Are you aware of any side effects of taking these drugs? 1. Yes 2. No 9. N/A

(If No, skip to Q21)

20. What are some of the side effects of taking these drugs? (Multiple correct answers are allowed)

1. Itching 2. Rashes 3. Swelling of parts of the body 4. Headache 5. Fever

6. Chills 7. Other, Specify………………………………………………….. 9.N/A

**21. If the drugs were given again, would you want it to be distributed the same way?**

1. Yes 2. No 8. Don’t Know 9. N/A

**22. How many times have these drugs been distributed in this community?** [ ] [ ] 99. N/A

**23. How many times have you taken these drugs yourself?** [ ] [ ] 99. N/A

**24. What suggestions do you have for improving future mass drug distribution exercises? …………………………………………………………………………………**

**…………………………………………………………………………………………**

**…………………………………………………………………………………………**

**…………………………………………………………………………………………**

**25. Do you know anyone with elephantiasis or a hydrocoele? 1. Yes 2. No (If No, skip to Q27)**

**26. Is there anyone in this household with any of the two conditions? 1. Yes 2. No 9. N/A**

**27. Is any treatment available to people with these conditions in this community?**

**1. Yes 2. No 8. Don’t know**

**28. What help is available for people with hydrocoeles from the health service?**

**......................................................................................................................................**

**………………………………………………………………………………………….**

**29. What help is available for people with elephantiasis from the health service?**

**…………………………………………………………………………………………**

**………………………………………………………………………………………….**

**………………………………………………………………………………………….**

**Thank you for spending time to answer these questions for me.**

**INTERVIEWER: ASK TO TALK WITH ALL THOSE WHO ARE PRESENT IN**

THE HOUSEHOLD AT THE TIME OF INTERVIEW (Head of household will answer for those not present)

| Name | Sex | Age in years | Currently Present Yes/No | Received Yes/No  (enter codes) # | Swallowed  Yes/No/ NA | No of tablets received | | Side Effects  (Enter Codes)* |
| --- | --- | --- | --- | --- | --- | --- | --- | --- |
|  |  |  |  |  |  | **Large** | **Small** |  |
|  |  |  |  |  |  |  |  |  |
|  |  |  |  |  |  |  |  |  |
|  |  |  |  |  |  |  |  |  |
|  |  |  |  |  |  |  |  |  |
|  |  |  |  |  |  |  |  |  |
|  |  |  |  |  |  |  |  |  |
|  |  |  |  |  |  |  |  |  |
|  |  |  |  |  |  |  |  |  |
|  |  |  |  |  |  |  |  |  |
|  |  |  |  |  |  |  |  |  |
|  |  |  |  |  |  |  |  |  |
|  |  |  |  |  |  |  |  |  |
|  |  |  |  |  |  |  |  |  |
|  |  |  |  |  |  |  |  |  |
|  |  |  |  |  |  |  |  |  |
|  |  |  |  |  |  |  |  |  |

#If answer for drug received is **No** put in one of the following codes

1. Less than 90cm 2. Pregnant 3. Chronic illness 4. Absent during treatment

4. Breast feeding 5. Other specify…………………..

*****Did anybody have any problem within two days after taking the drug?

1. No problem 2. Fever 3. Headache 4. Vomiting 5. Myalgia

6. Dizziness 7. Itching 8. NA 9. Diarrhoea 10. Other specify…………….
